# A Pilot Study on Management Practices in Dairy Farms in the Basque Country: Focus on Colostrum Feeding and Vaccination

**DOI:** 10.3390/ani15091336

**Published:** 2025-05-06

**Authors:** Maddi Oyanguren, Elena Molina, Maitane Mugica, Ainara Badiola, Ana Hurtado, Gorka Aduriz, Natalia Elguezabal

**Affiliations:** Animal Health Department, NEIKER, Basque Institute for Agricultural Research and Development, Basque Research and Technology Alliance (BRTA), E-48160 Derio, Bizkaia, Spain; moyanguren@neiker.eus (M.O.); emolina@neiker.eus (E.M.); mmugica@neiker.eus (M.M.); abadiola@neiker.eus (A.B.); ahurtado@neiker.eus (A.H.); gaduriz@neiker.eus (G.A.)

**Keywords:** colostrum, passive immunity transfer, immunoglobulin, T lymphocytes, antibiotics, vaccination

## Abstract

Colostrum feeding is crucial for calf rearing to guarantee passive immunity transfer (PIT) of antibodies. The present study aimed to evaluate the effect of different management practices on the calf’s immunological parameters focusing on vaccination and colostrum management. Data were gathered on management routines, vaccination programs, and antimicrobial usage. Farmers were provided with colostrum management guidelines and a digital Brix refractometer to enhance colostrum feeding practices. Colostrum quality, PIT and lymphocyte subpopulations in both colostrum and blood were analyzed for further characterization. The combined farm and laboratory data were then examined to evaluate each farm’s situation. Farmers reliably monitored colostrum quality by Brix refractometry and were able to modify colostrum management in a way that favored PIT. High-quality colostrum was linked to better PIT outcomes. Notably, Farm C, the sole non-vaccinated farm, reported higher antibiotic usage in both calves and lactating animals and showed reduced γδ T cell levels in colostrum. In conclusion, lymphocyte subpopulation content should be further studied as a trait of colostrum quality as well as of PIT. Failure to implement a vaccination program in the farm can have negative consequences on colostrum quality, as shown when analyzing both immunoglobulins and lymphocytes. This can result in a higher number of antibiotic treatments that may in turn be followed by different patterns of antimicrobial resistance.

## Simple Summary

Ensuring the health of calves is fundamental to having productive adult dairy cattle. Calf health heavily relies on colostrum intake, as newborns are immunologically naïve at birth. This study evaluated the impact of different colostrum management practices and the implementation of vaccination programs on calf immune parameters and antimicrobial usage across all ages. Differences in colostrum handling and farm operations led to variability in the success of passive immunity transfer among farms. As expected, high-quality colostrum was correlated with successful passive immunity transfer. The absence of vaccination programs had a negative effect on colostrum and resulted in an increase in antimicrobial use in animals of all ages, underscoring the importance of vaccinations for maintaining calf and herd immunity.

## 1. Introduction

Colostrum feeding is crucial for calf rearing because there is no transfer of immunoglobulins (Igs) from the dam to the embryo-future calf during gestation [1]. Hence, bovine neonates lack antibodies at birth, as they are immunologically naïve [2]. Their immunocompetence in early phases greatly depends on the passive immunity transfer (PIT) of antibodies through colostrum intake from their dams. Igs have been considered the most important component of colostrum, especially IgG, which accounts for 85–90% of total Ig in bovine colostrum [2]. In addition to antibodies, colostrum contains a mixture of cytokines, growth factors, hormones, immune cells, and nutrients [3] that guarantee calves are nurtured and hydrated in their first days of life. Finally, colostrum feeding can have a later impact on the productive life of the animal since there is evidence that neonatal serum immunoglobulin concentration can be positively correlated with milk production during the first lactation [4]. Therefore, correct colostrum ingestion secures both health, growth, and productive life.

In-farm colostrum quality monitoring based on measuring Ig helps producers to quickly identify and correct problems in the colostrum management practices [5]. Despite information regarding correct colostrum management to ensure PIT, sometimes there is a failure of passive immune transfer (FPIT) [6]. FPIT contributes to calf morbidity and mortality [7], since bovine neonatal diarrhea and bovine respiratory disease incidence have increased [7], reductions in weight gain are observed [8], and lower milk yield in the productive future has been reported [9].

Vaccinating the dam has been shown to elevate the concentrations of specific immunoglobulins, boosting colostrum’s protective efficacy [10,11,12]. Administering vaccines pre-partum to strengthen maternal immunity and promote the transfer of higher levels of specific antibodies to offspring via colostrum is a promising strategy currently under extensive investigation.

In addition to Ig, colostral leukocyte transfer and absorption through the gut has been described in neonates [13], showing their influence in the immune response [14]. More specifically, the absorbance of maternal immune cells has been related to the reduction in diarrhea caused by *Escherichia coli* [15] and has a better response against bovine viral diarrhea [16]. The transfer of maternal leukocytes through colostrum in goats has been demonstrated to impact the early adaptive immune responses in newborns [17].

The Basque Country is a region located in the Northern part of Spain, where dairy cattle production is one of the largest sectors of agriculture. Dairy calf management is performed intensively, and calves are separated from the dams immediately after birth. Farmers are responsible for colostrum feeding to calves and should follow guidelines that ensure this process. Improving calf health status through improved colostrum management, hygiene, and vaccination should help decrease infectious diseases and antimicrobial use.

The present pilot study aimed to evaluate and associate the effects of different management practices on colostrum quality and the calf’s immunological parameters (PIT, IgGs, lymphocyte subpopulations), focusing on vaccination and colostrum management.

## 2. Materials and Methods

### 2.1. Study Design

This observational study was performed within the framework of a larger study on five farms (F1–F5), the main characteristics of which are described elsewhere [18]. The study was conducted on four dairy cattle farms (A–D) in the Basque Country (a region of the North of Spain). The matching farms between the studies are A (F1), B (F5), C (F3), and D (F4). Before the study started on each farm, the veterinary clinician and farmer completed a questionnaire addressing farm information regarding general management practices and vaccine programs in a face-to-face interview with one of the authors from this study. On this day, farmers were supplied with a digital refractometer and trained on how to operate it, with different documents for data collection and a colostrum management guide.

For this study, 7–10 calvings of female births were sampled at each farm. Farmers were asked to follow management guidelines (Supplementary Information S1 and Table S1), including measuring colostrum IgG content by digital refractometer, annotating Brix% values, and increasing colostrum volume intake depending on the obtained Brix% values. Colostrum samples for analysis should be collected as near to calving and first feeding as possible by the farmers, whereas blood samples were collected 24–48 h after parturition and at 3 months of age by a veterinary clinician. Farmers collaborated with the veterinary clinician to obtain samples, estimating IgG concentration in colostrum by digital refractometry and annotating Brix% values. All samples were sent to the laboratory for posterior analysis.

**Figure 1:**
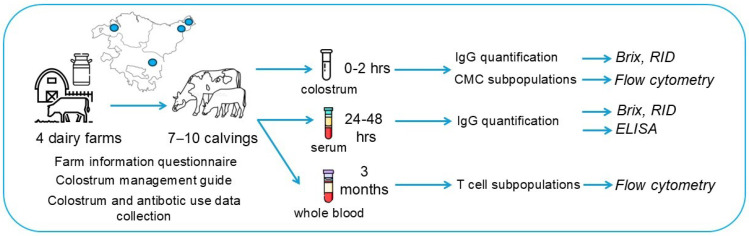
Schematic representation of the study design.

Finally, farmers should share all antibiotic treatments administered on the farm during the study period (one year—February 2019–February 2020) since these are registered by law (RD 191/2018, 6 de abril, Electronic transmission of data for veterinary antibiotic prescriptions intended for food-producing animals for human consumption (*Transmisión electrónica de datos de las prescripciones veterinarias de antibióticos destinados a animals productores de alimentos para consumo humano*). These should be verified by the veterinary clinicians and shared with researchers. A schematic representation of the study design is shown in Figure 1.

The blood sampling was excluded from the scope of application of RD 53/2013 [19] the protection of experimental animals, since it complied with point (b) of article 2, that is, samples were taken through non-experimental clinical veterinary practices from healthy Holstein Friesian calves of commercial dairy farms located in the Basque Country.

### 2.2. Colostrum and Serum IgG Measurement

#### 2.2.1. In-Farm

Colostrum samples were taken nearest to the first 2–4 h after birth. Brix refractometry was used to estimate IgG concentration and adapt feeding volumes when possible. Samples were stored refrigerated at 4 °C until they were sent to the laboratory the next day. Blood samples for IgG quantification in serum were drawn in tubes without anticoagulant (Becton Dickinson BD ™, Plymouth, UK) by venipuncture with 20 G needles (Becton Dickinson BD ™, Plymouth, UK) 24–48 h after the first colostrum feeding in tubes with silica particles as a clot activator and sent immediately to the lab.

#### 2.2.2. In-Lab

Once the blood samples arrived, these were centrifugated at 1000× *g* for 20 min at room temperature (RT), and the serum was collected. IgG estimation by Brix (Hi96800, Hanna Instruments, Asheville, Raleigh, NC, USA) was performed on both serum and colostrum samples, and aliquots were frozen at −20 °C until the RID assays were performed. For RID, samples were allowed to thaw at RT, and a commercial kit (IBIGG10EN^®^, GTP Bioways, Touluse, France) was used. 50 μL of colostrum were diluted at 1/500 in 3 steps and 50 μL of serum was diluted at 1/200 in two steps. Samples were deposited in the RID agar chamber wells along with reference samples supplied by the kit. Plates were incubated in a humid box for 18 h at RT. The following day, plates were filled with acetic acid 2% and incubated for 1 min at RT. Acetic acid was drained and agar gel was rinsed twice with distilled water. The plate was filled with distilled water one last time and incubated for 10–15 min at RT. Once this incubation finished, the diameter (mm) of the precipitating rings was measured. To obtain the IgG concentration (μg/mL) of each colostrum sample, a linear regression was calculated with the ring diameter values and standard curve concentration values. A high-quality colostrum is considered to contain over 50 mg/mL of IgG [20].

### 2.3. Analysis of Lymphocyte Subpopulations of Colostrum and Peripheral Blood

#### 2.3.1. Peripheral Blood Mononuclear Cell (PBMC) Isolation

Blood sampling was performed on all calves at 3 months of age in EDTA tubes (Becton Dickinson BD ™, Plymouth, UK) by venipuncture with 20 G needles (Becton Dickinson BD ™, Plymouth, UK) by a veterinary clinician. Once at the laboratory it was transferred to 50 mL tubes where hypotonic lysis was performed with 30 mL of sterile distilled water. The lysis was stopped by adding 3 mL of NaCl 10%. After centrifugation at 300× *g* for 10 min at RT, the solution was resuspended in 7 mL of PBS and layered over 3 mL of Histopaque 1077 (Millipore-Sigma®, St. Louis, MO, USA). After a centrifugation step of 700× *g* for 35 min at RT without deceleration, the layer containing PBMCs was aspirated. Subsequently, two washes were performed with HBSS (Corning®, Corning, NY, USA) and centrifugations at 300× *g* for 10 min at RT. PBMCs were stored in RPMI (Gibco, Grand Island, NY, USA) (47%)-FBS (HyClone™ Cytiva, Malborough, MA, USA) (42.5%)-DMSO (Millipore-Sigma®, St. Louis, MO, USA) (10%) Penicillin-Streptomycin (Gibco, Grand Island, NY, USA) (0.5%) at −80 °C until cytometer analysis.

#### 2.3.2. Colostrum Mononuclear Cell (CMC) Isolation

Colostrum was centrifugated at 4 °C for 15 min at 600× *g* to remove the lipid layer and the supernatant. Subsequently, two washes were performed with 10 mL of PBS-FBS (1%) (HyClone™ Cytiva, Malborough, MA, USA) (1%)-Azide (Merck Millipore, Burlington, MA, USA) (0.01%) centrifugating at 600× *g* for 10 min at 18 °C. The supernatant was removed, and the pellet was resuspended in 7 mL of PBS-FBS (1%)-Azide (0.01%). Afterward, the solution was layered over 7 mL of Histopaque 1119 (Millipore-Sigma®, St. Louis, MO, USA) and centrifugated at 900× *g* for 30 min at RT. The floating CMC-containing layer was recovered and washed twice with 10 mL of PBS-FBS (HyClone™ Cytiva, Malborough, MA, USA) (1%)-Azide (Merck Millipore, Burlington, MA, USA) (0.01%) 300× *g* for 10 min at RT. CMCs were frozen in RPMI (Gibco, Grand Island, NY, USA) (47%) SBF (Marlborough) (42.5%)-DMSO (Millipore-Sigma®, St. Louis, MO, USA) (10%), Penicillin-Streptomycin (Gibco, Grand Island, NY, USA) (0.5%) and stored at −80 °C until cytometry analysis.

### 2.4. CMC and PBMC Subpopulation Analysis

Firstly, CMCs and PBMCs were thawed with 2 mL of FBS (HyClone™ Cytiva, Malborough, MA, USA) and washed twice with HBSS (Corning®, Corning, NY, USA) 300× *g* for 8 min at RT. The cell pellet was resuspended in PBS-FBS 2% and counted. CMCs and PMBCs were centrifuged at 300× *g* 8 min RT and resuspended in 800 μL PBS-FBS (HyClone™ Cytiva, Malborough, MA, USA) 2% and split into 4 tubes of 200 μL. Subsequently, tubes were incubated with 1 μL of each of the following antibodies, mouse anti-bovine CD4+ FITC (0.005 μg/μL), mouse anti-bovine CD8+ FITC (0.005 μg/μL), and mouse anti bovine WC1 γδ FITC (0.0025 μg/μL l) (BIO RAD™, Hercules, CA, USA), for 30 min at 4 °C. Afterwards, tubes were centrifuged at 300× *g* for 8 min and resuspended in 200 μL PBS-FBS (HyClone™ Cytiva, Malborough, MA, USA) 2%. These tubes were divided into two with 100 μL in each to have samples stained with Hoescht 33258 (Invitrogen^™^, Thermo Fisher Scientific, Waltham, MA, USA) at 0.0025 mg/mL at 4 °C for 30 min and unstained to assess viable cells.

Cell suspensions were analyzed using a CytoFlex Flow Cytometer (Beckman Coulter^®^, Indianapolis, IN, USA). The total lymphocyte population was identified according to their specific FSC/SSC patterns. Live-Dead cells were determined by Hoescht 33258 (Invitrogen^™^, Thermo Fisher Scientific, Waltham, MA, USA) through the Pacific Blue Channel. Viable lymphocyte subpopulation percentages were expressed as the number of FITC (CD4+, WC1 γδ T cells) or APC (CD8+) positive cells. Flow cytometry data were analyzed with CytExpert v2.3 software (Beckman Coulter^®^, Indianapolis, IN, USA).

### 2.5. Data Analysis and Statistics

The chi-square test was used to compare antibiotic treatment administration among farms. Correlation analysis was performed by Pearson: between colostrum IgG concentration measured in farm and laboratory, Brix and RID measurements of colostrum and serum IgG, PIT achievement and high-quality colostrum (HQC) and CD8 population levels between colostrum and blood. Correlations were interpreted as: very strong: 0.8–1, strong 0.6–0.8, moderate: 0.4–0.6 and weak: 0.2–0.4. For HQC (threshold at 50 mg/mL) and PIT achievement classification the recommendations by Lombard and colleagues [20] were followed. This system defines four categories based on serum IgG concentration, intending to have 40% of calves in the “excellent” category, 30% in “good”, 20% in “fair”, and less than 10% in “poor”. Colostrum and serum IgG concentrations obtained by RID were calculated using linear regression and multiplying by the corresponding dilution factor after.

Comparison of parameters among farms was performed by Kruskal–Wallis and post hoc Dunn’s multiple comparison test (IgG in serum, CD4, CD8, and γδ WC1 cell percentages). Comparison of parameters between vaccinated (VAX) and non-vaccinated farms (NO VAX) was performed using *t*-test (CD4/CD8 ratio from blood and colostrum) or Mann–Whitney test (CD4, CD8, and WC1 cell percentages from blood and colostrum).

All analyses were performed with Microsoft Excel (Version 2409 Build 16.0.18025.20160) and Graph Pad Prism 9 statistical software (9.5.1). A *p*-value of less than 0.05 was considered significant.

## 3. Results

### 3.1. Management Practices and Vaccination Differences Between Farms

After completing a questionnaire on management practices and vaccination programs in face-to-face interviews with the farmers and veterinary clinicians, all the information was compiled. Colostrum feeding and dry period management practices are detailed in Table S2, and vaccination programs are specified in Table S3.

The greatest difference between farms may be summarized in the following: Farm C did not vaccinate at all, while the rest of the farms vaccinated against at least three diseases; Farm B implemented selective antimicrobial dry cow therapy (DCT) and fed calves with farm milk until weaning, while the rest applied blanket dry therapy and feed with milk replacer; Farm C always feeds calves with colostrum from their dam, while the rest fed from multiparous or recent calving dams; Farms B and D milked colostrum shortly after parturition, while A and C extracted colostrum at ordinary milking time; and, Farm A had the largest number of milking cows, i.e., 200 compared to 75, 100, and 120 on Farms B, C and D, respectively.

When supplying the colostrum management guidelines (Supplementary File Information S1) and the Brix refractometer all farmers committed themselves to record colostrum Brix% and try to adjust the volume based on the results. They would also aim to feed colostrum during the first 6 h after birth.

### 3.2. Reported Antibiotic Treatments

Farmers were asked to register all antibiotic treatments during the study period (one year) and share the data with researchers, which they did except for farm B. Antibiotic treatments reported in animals under one year and in lactating animals are detailed in Table 1. Mastitis and lameness were the main diseases for which antibiotic treatment was administered to cows in lactation. Farm C reported a significantly greater percentage of treatments in both analyzed groups: heads under one year of age (X^2^ = 9.94, *p* = 0.0069) and heads in lactation (X^2^ = 63.44, *p* < 0.0001) compared to the other farms when compared to the other farms.

### 3.3. Colostrum IgG Estimation with Brix Refractometer

To verify that farmers correctly used the digital refractometer for colostrum quality assessment, in-farm and in-lab results from Brix% values were analyzed finding a very strong positive correlation (Pearson 0.841; *p* < 0.0001, n = 30). When Brix% values were separately analyzed for each farm, strong correlations were found on farms A, C, and D as detailed in Table 2.

### 3.4. Refractometry and RID Correlation for IgG Estimation in Colostrum

RID is the gold standard method for IgG concentration quantification in colostrum but it must be performed in the laboratory, whereas Brix refractometry provides an estimation but can be used in-farm. To assess if refractometry could be of value for farmers in this small cohort, Brix% values and RID values were analyzed finding a strong positive correlation (Pearson 0.674; *p* < 0.0001, n = 32).

### 3.5. Colostrum Quality Among Farms

**Figure 2:**
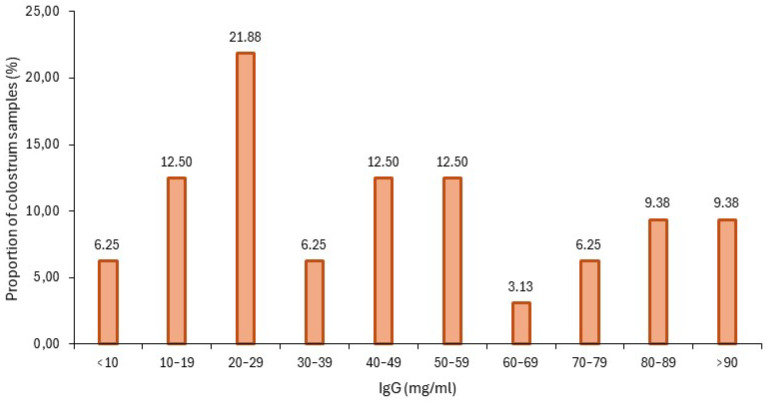
Bar plot with the distribution of IgG concentration (mg/mL) in the analyzed colostrum samples.

Colostrum quality was evaluated by estimating and measuring the colostral IgG concentration by refractometry and RID, respectively. The distribution of IgG concentration in the analyzed colostrum samples is detailed in Figure 2. Colostrum was classified as high-quality (>50 mg/mL) or not, considering the RID values. The percentage of HQC samples for each farm is detailed in Table 3. The colostrum samples with the highest quality were found in Farm B, whereas the lowest belonged to Farm A.

### 3.6. Passive Immunity Among Farms

**Figure 3:**
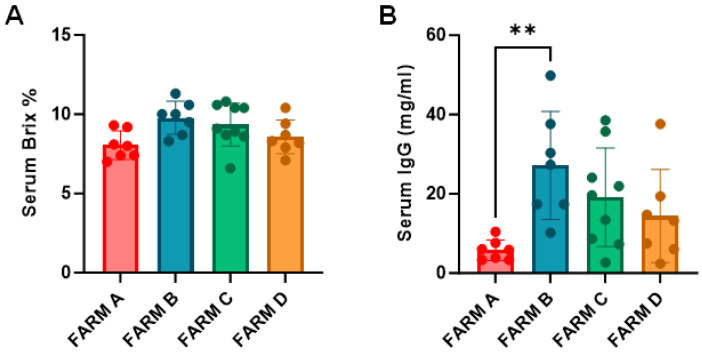
Passive immunity transfer estimation in farms. (**A**) Brix% in serum, (**B**) IgG in serum measured by RID. Mean bar plots with individual values in dots, and standard deviation. Kruskal–Wallis with Dunn’s post hoc was performed to compare among farms. ** *p* < 0.01.

The concentration of IgG in serum at 24–48 h of birth varied among calves and is detailed in Supplementary Table S4. Following the classification determined by Lombard and collaborators [21] calves were allocated into four categories: excellent (>25 mg/mL), good (17.9–24.9 mg/mL), fair (10–17.9 mg/mL), and poor (<10 mg/mL). Categorization data along with the percentage of PIT and FPIT for each farm are detailed in Table 4. Although the overall PIT was accomplished in over 50% of the cases considering data from all farms, only Farm B accomplished the goal set by Lombard and collaborators, with 50% of calves belonging to the excellent category and less than 10% to the poor category. Farm A contributed with the worst results and when Brix% and RID values among farms were compared, significant differences were found between Farm A and B for RID values (Figure 3B).

**Figure 4:**
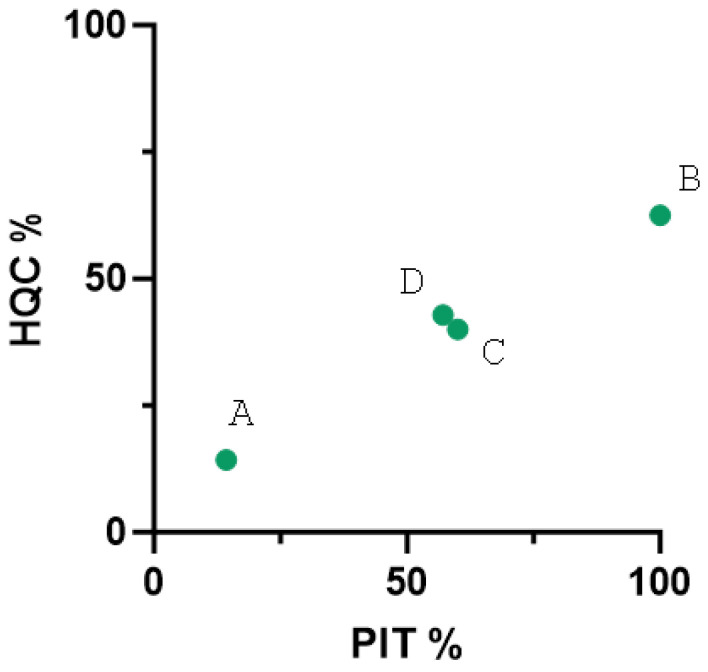
Correlation plot between high-quality colostrum percentage and passive immunity transfer percentage in farms. Each dot is labeled and represents the intersection of both values for each farm (Pearson 0.993, *p* = 0.0064, n = 4).

HQC on the farm is one factor affecting PIT achievement. The farm percentage of HQC and the farm percentage of successful PIT for each farm were analyzed and plotted (Figure 4) finding a very strong positive correlation (Pearson coefficient = 0.993, *p* = 0.0064, n = 4).

### 3.7. Refractometry and RID Correlation for IgG Estimation in Serum

For this purpose, in the lab the reliability of the Brix refractometer was evaluated for serum IgG estimation, finding a strong correlation between Brix% and RID values for serum samples (Pearson coefficient of 0.686 *p* < 0.0001, n = 32).

### 3.8. CD4, CD8 and γδ T Cell Levels in Colostrum and Blood

Both colostrum and blood lymphocyte subpopulations were analyzed by flow cytometry. Although a high variability was found between animals (Table 5), CD4+ T cells were predominant in colostrum, followed by WC1 γδ T cells and CD8+ T cells. Instead, WC1 γδ T cells were predominant, followed by CD4+ and CD8+.

Farm C had significantly higher CD8 T cells levels in colostrum compared to Farm B (*p* = 0.016). In blood, Farm B had a significantly higher level of CD4+ T cells compared to Farm A (*p* = 0.003) and Farm C had significantly higher levels of CD8+ T cells compared to Farm A (*p* = 0.019).

### 3.9. CD4, CD8 and γδ T Cell Levels in Non-Vaccinated Animals

**Figure 5:**
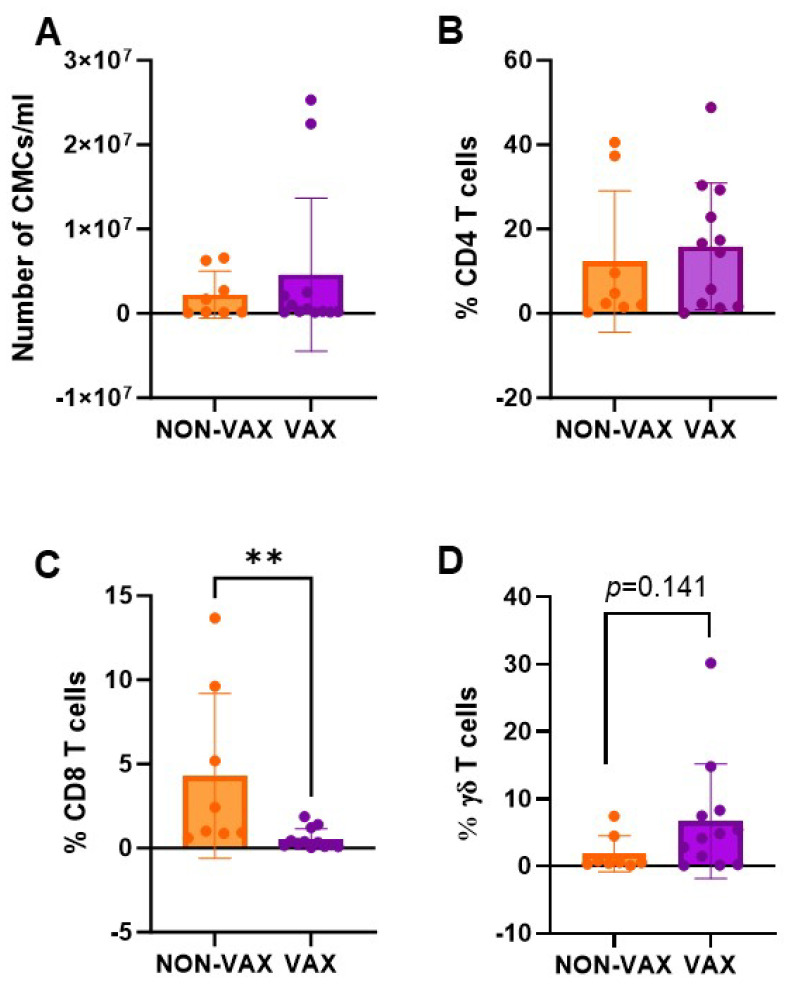
Colostrum mononuclear cell analysis comparing non-vaccinated (NON-VAX) and vaccinated (VAX) farms. (**A**) CMCs/mL of colostrum, (**B**) CD4 T cell percentages, (**C**) CD8 T cell percentages, and (**D**) γδ T cell percentages. Mean bar plots with individual values in dots, and standard deviation. Mann–Whitney’s test was performed to compare among groups. ** *p* < 0.01.

**Figure 6:**
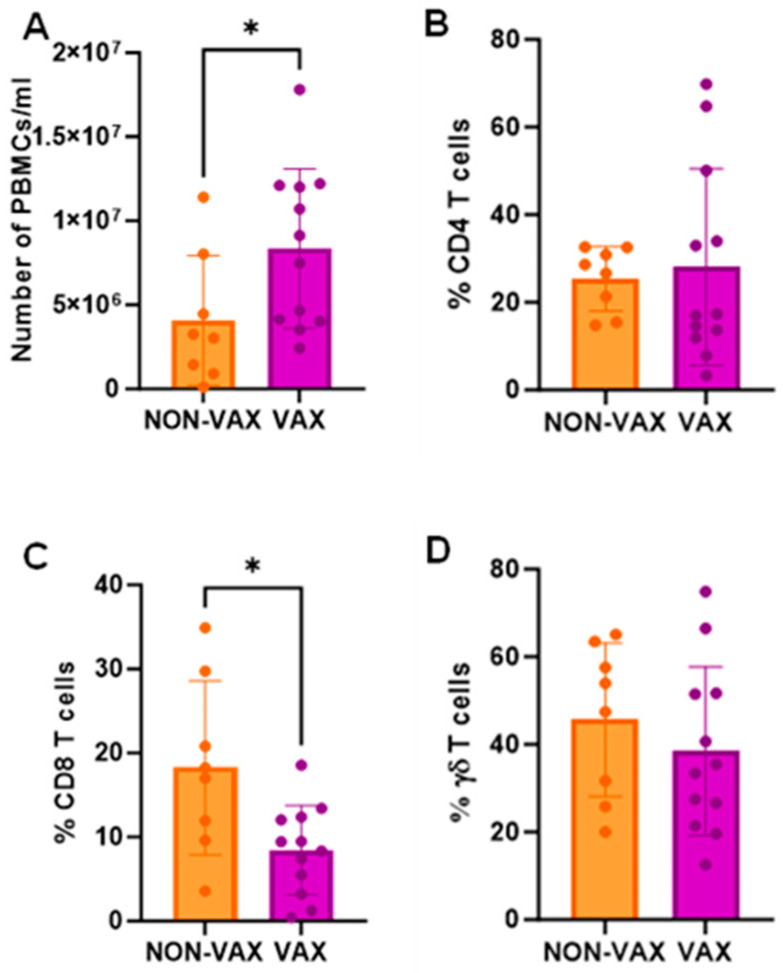
Peripheral blood mononuclear cell analysis comparing non-vaccinated (NON-VAX) and vaccinated (VAX) farms. (**A**) PBMCs/mL of colostrum, (**B**) CD4 T cell percentages, (**C**) CD8 T cell percentages, and (**D**) γδ T cell percentages. Mean bar plots with individual values in dots, and standard deviation. Mann–Whitney’s test was performed to compare among groups. * *p* < 0.05.

Differences were observed in cell populations of colostrum and blood when animals from Farms A, B, and D were gathered in one group (VAX: vaccinated farms) and compared with animals from Farm C (NON-VAX: non-vaccinated farm). CD8 T cell levels were significantly higher in colostrum among the non-vaccinated animals (Figure 5C) and, a trend towards a lower percentage of γδ T cells in colostrum from non-vaccinated animals was also observed (*p* = 0.141) (Figure 5D). Although the total number of PBMCs/mL in blood was significantly lower in the non-vaccinated animals (Figure 6A), the CD8 T cell levels were significantly higher (Figure 6C).

## 4. Discussion

The health and welfare of dairy calves are crucial for the animal’s early development and for the long-term profitability of the farm. Since neonate calves have an immature immune system, they must rely on colostrum intake until their immune system develops and matures.

For proper colostrum management information on colostrum quality is vital. Although RID is considered the gold standard technique for colostrum and serum IgG quantification [22,23], it is not attractive for farmers as it requires having laboratory equipment and is time-consuming [22,24]. Previous studies have described the Brix refractometer as a reliable tool for IgG estimation in colostrum [23,24], and serum [22,25]. In this study, in-farm and in-lab Brix values of colostrum showed a very strong correlation demonstrating that farmers can easily use the refractometer. It would also be desirable to estimate PIT to enable close follow-up of animals with FPIT. In this study, correlations between in-lab Brix and RID values were strong, although slightly lower than those found in previous studies for colostrum [23,24] and serum [22,26]. These differences are probably due to the lower sample size in our study and should not discourage farmers from using refractometry.

Colostrum quality varied between farms, with Farm B having the best colostrum quality and showing the greatest differences with Farm A. Colostrum quality depends on parity, genetics, general health, vaccination status, and the length of the dry period [27] but also on the colostrum milking time. Colostrum quality is best when collected 1–2 h after calving [27] and a delay in colostrum milking is associated with a decay of quality [28]. Comparing Farm A and B management practices brings forward three main differences related to colostrum milking time, feeding strategy at weaning, and dry period length. Farm A collected colostrum at ordinary milking time instead of shortly after parturition (Farm B), fed milk replacer instead of farm milk, and had a longer dry period (over 45 days). The delay in colostrum collection could be affecting Farm A’s colostrum quality, and the variable length of the dry period may affect how and when antibodies reach colostrum during colostrogenesis. Farm B’s strategy of feeding calves farm whole milk instead of milk replacer, like the rest of the farms, may also be improving its colostrum quality. Some studies have stated that animal performance improves when whole milk is fed [29,30]. Other factors that may directly or indirectly affect colostrum quality could be herd size (Farm A is larger, meaning heavy work) and DCT (Farm A used blanket antimicrobial treatment, meaning more antibiotic use).

Farm C reported the highest number of antibiotic treatments in both animal age groups (under one year and lactating animals). The non-use of vaccines at Farm C could explain the increase in antimicrobial use as vaccines can provide herd protection and, in some cases, a certain degree of heterologous protection [31]. In normal circumstances, dams produce antibodies against the major pathogens that circulate in the farm, and, if vaccinated, dams will also produce antibodies against the specific pathogens the vaccine was designed against. If vaccination takes place at the correct time during gestation, these antibodies will be secreted into colostrum conferring passive specific protection to their calves through colostrum ingestion. Furthermore, many of the vaccines applied in Farms A, B, and D include the adjuvant hydroxide aluminum which has shown trained immunity mechanism activation [32]. Aluminum hydroxide induces differentiation of monocytes into dendritic cells, activates innate immunity pathways triggered by pattern recognition receptors (PRRs), and activates NLPR3 inflammasome [33]. Farm A was the farm with the second highest percentage of reported antimicrobial treatments in both animal groups despite the vaccination program, it had the lowest quality colostrum and the worst PIT, so it is expected that calves would not be fully protected, at least in early phases. Farm C had the second worse colostrum quality. Farms A and C share the fact that colostrum milking was performed at ordinary milking time rather than shortly after parturition, which would imply a delay, at least for several animals. However, PIT in Farm C is the second best, probably because the volume of colostrum fed to new-born calves was increased based on the Brix measurements as recommended.

Colostrogenesis begins one month before calving and finalizes after calving [34], meaning that colostrum production occurs exclusively during the dry period. The dry-off period should not only be considered a time for the cow’s udder to rest and recover, but also to prepare the cow’s immune system to produce high-quality colostrum with specific antibodies that will protect the calf after birth. Pre-partum vaccination to increase maternal immunity and generate antibodies is gaining relevance. Many studies have shown that immunization during this period has a positive effect on the transfer of passive immunity of antibodies to calves [10,11,12] and even cell-mediated immune transfer can be enhanced by maternal vaccination [16]. Vaccines were not used on Farm C and animals would therefore lack the antibodies that would protect them against infections that could be behind the increase in antibiotic treatments.

When IgG quantity was analyzed based on vaccination (VAX: Farms A, B, and D vs. NON-VAX: Farm C), significant differences were not detected. In contrast, Dunn et al. found that farms that immunized against *Salmonella* had greater IgG concentrations in colostrum than those that did not vaccinate [35]. Although the study sample could influence our result, we should consider that the effects would not only reflect the amount of total IgGs, but more importantly the amount of IgGs against the specific pathogens that the animals are in contact with at that moment.

However, differences were detected in cell subpopulations when the inclusion of a vaccination program was considered. CD8 T cell percentage was elevated in both colostrum and blood in Farm C compared to the rest of the farms. CD8-positive T cells in colostrum produce IFN-γ [36] and have been suggested as important players in passive cellular immunity in human breast milk [37], although data on bovine colostrum or milk is lacking. Their presence is therefore desirable in the colostrum and for some unknown reason Farm C had higher levels. On the other hand, colostrum from Farm C presented lower levels of γδ T cells. γδ T cells are known to recognize non-peptide antigens without MHC restriction [38] and play an important role in protection until the immune system of the calf is fully matured [39]. Colostrum deprived calves show decreased γδ T-cell percentages in blood during the first weeks of life [40]. This could also explain the higher antibiotic treatment rate in Farm C’s young animals, assuming calves did not receive enough γδ T-cells through colostrum. In any case, calves at 3 months of age from Farm C showed γδ T-cells levels in blood comparable to the rest of the farms, meaning that although poor lymphocyte transfer through colostrum would have happened, this can be reverted afterward. Having data from T cell subpopulations in calf blood shortly after colostrum ingestion would have clarified this. Given the gaining relevance of dam lymphocyte transfer through colostrum for the calf to acquire immunocompetence [17] and stimulate intestinal immunity [2], future research on lymphocyte content to define colostrum quality and PIT should be considered. Total PBMC count was lower in Farm C and was not further investigated; it could be due to a lower number of B cells caused by the lack of vaccination in the farm, although 3 months is enough time to have led to early antigen encounters that should have elevated this lymphocyte subset.

Antibiotic resistance profiles of some zoonotic and indicator bacteria isolated from the farms included in this study have been described elsewhere [18,41,42]. Isolation frequency of cefotaxime-resistant *E. coli* was significantly lower in Farm A (lactating cows, heifers and calves) compared with Farms B, C, and D [18]. Overall, isolation frequency was lower in heifers compared to the other age groups, except for Farm C, which had a remarkably higher isolation frequency in heifers. When zoonotic campylobacters were investigated, *Campylobacter jejuni* was isolated from all farms, while *Campylobacter coli* was only isolated from Farms B and D [41]. In this case, Farm C had the lowest rate of multi drug resistance (MDR) among isolated strains compared to other farms, although a strain isolated from calves in this farm was MDR and the rate of susceptible strains was lower [41]. The largest diversity of antimicrobial resistance (AMR) was found in farms B and D [41]. When looking into *Enterococcus*, a MDR *Enterococcus faecalis* strain resistant to five antibiotics was isolated from lactating cows in Farm C and isolates resistant to four antibiotics in Farms B and D [42]. Overall, these findings indicate that AMR is widespread and although there was a higher percentage of reported antibiotic treatments in Farm C the rest of the farms also harbor MDR bacteria.

## 5. Conclusions

In conclusion, this pilot study shows that farmers can reliably monitor colostrum quality by Brix refractometry and adapt feeding to increase PIT. High quality colostrum is correlated to PIT and although PIT success is traditionally measured by Ig content in serum, future research should focus on lymphocyte subpopulation content and evaluate the use of this parameter as a trait of colostrum quality. This small study sample shows that not vaccinating the herd may have negative consequences on colostrum quality, based both on immunoglobulin and leukocyte content. Not achieving immunocompetence based on cellular immune response in early ages can result in a higher number of antibiotic treatments on the farm at all ages, and this can in turn affect AMR.

## Figures and Tables

**Table 1:** Reported antibiotic treatments during the study period.

Under One Year					
Farm	Number of Non-Milked Heads	Number of Treatments	Number of Treated Heads		% Treated/Non-Milked Heads
A	110	6	6		5.45
C	140	20	20		14.28
D	130	6	6		4.61
**In Lactation**					
**Farm**	**Number of Milked Heads**	**Condition**	**Number of Treatments**	**Number of Treated Heads**	**% Treated/Milked Heads**
A	200	Mastitis	124	49	24.5
		Lameness	115	50	25
		Respiratory	1	1	0.5
		Total	240	100	50
C	100	Mastitis	54	36	36
		Lameness	32	32	32
		Respiratory	3	3	3
		Total	89	71	71
D	120	Mastitis	31	20	16.66
		Lameness	NR	NR	NR
		Respiratory	2	2	1.66
		Total	33	22	18.33

NR—not registered.

**Table 2:** Correlation coefficients between the in-farm and in-lab Brix% values obtained on each farm.

Farm	Pearson	*p*	n
A	0.986	<0.0001	7
B	0.661	0.106	7
C	0.916	0.0005	9
D	0.854	0.0145	7
Total	0.841	<0.0001	30

**Table 3:** Colostrum quality based on Brix, RID measurements and percentage of high-quality colostrum in each farm and in the entire study.

Colostrum Quality				
Farm	Brix% Mean (std.)	IgG (mg/mL) Mean (std.)	HQC%	n
A	20.24 (2.82)	32.26 (14.89)	14.29	7
B	21.13 (5.88)	53.09 (33.40)	62.50	8
C	18.77 (6.61)	45.17 (28.84)	40.00	10
D	20.71 (7.25)	47.15 (32.35)	42.86	7
Total	20.21 (5.75)	44.76 (28.17)	40.63	32

std.: standard deviation; HQC: High-quality colostrum.

**Table 4:** PIT classification of calves based on their serum IgG concentration measured by RID and percentage of successful PIT and FPIT obtained in each farm and overall.

Farm	Excellent (>25 mg/mL)	Good (17.9–24.9 mg/mL)	Fair (10–17.9 mg/mL)	Poor (<10 mg/mL)	PIT	% PIT	% FPIT	n
A	0	0	1	6	1	14.29	85.71	7
B	4	1	3	0	8	100.00	0.00	8
C	2	3	1	4	6	60.00	40.00	10
D	1	1	2	3	4	57.14	42.86	7
Total	7	3	8	13	19	59.38	40.62	32

**Table 5:** Colostrum and blood lymphocyte CD4, CD8 and WC1 γδ T percentage levels in each farm and for all animals included in the study.

	Colostrum			Blood			
Farm	CD4 Mean (std.)	CD8 Mean (std.)	WC1 Mean (std.)	CD4 Mean (std.)	CD8 Mean (std.)	WC1 Mean (std.)	n
A	16.43 (21.8)	0.42 (0.46)	5.35 (6.10)	10.74 (5.29)	4.59 (3.59)	38.35 (21.08)	5
B	12.44 (15.66)	0.11 (0.07)	10.61 (16.96)	56.20 (19.40)	11.75 (2.06)	22.57 (3.65)	3
C	12.31 (16.71)	4.29 (16.7)	1.83 (2.67)	25.36 (7.31)	18.24 (10.34)	45.63 (17.55)	8
D	17.83 (3.53)	1.03 (0.73)	5.35 (2.26)	28.74 (16.39)	10.82 (6.33)	50.67 (17.48)	4
All	14.46 (15.39)	2.04 (3.55)	4.73 (7.13)	27.01 (17.71)	12.37 (8.94)	41.36 (18.46)	20

## Data Availability

The data presented in this study is available on request from the corresponding author.

## Supplementary Materials

The following supporting information can be downloaded at: https://www.mdpi.com/article/10.3390/ani15091336/s1, Information S1. Colostrum management guidelines. Table S1. Colostrum total volume feeding adaptation in the first 12 hours and the successive feedings depending on the values obtained by Brix refractometry. Table S2. Colostrum, feeding, and dry period management in farms. Table S3. Vaccination programs run on farms. Table S4. IgG estimation in serum.

## Abbreviations

The following abbreviations are used in this manuscript:

PIT	Passive immunity transfer
Ig	Immunoglobulin
FPIT	Failure of passive immune transfer
RID	radioimmunodiffusion
RT	Room temperature
PBMC	Peripheral blood mononuclear cell
CMC	Colostrum mononuclear cell
HQC	High-quality colostrum
DCT	Dry cow therapy
NON-VAX	Non-vaccinated farm
VAX	Vaccinated farms
MDR	Multi drug resistance
AMR	Antimicrobial resistance

## References

[1] Peter A.T. (2013). Bovine Placenta: A Review on Morphology, Components, and Defects from Terminology and Clinical Perspectives. Theriogenology.

[2] Barrington G.M., Parish S.M. (2001). Overview of Immunologic Development in Utero. Vet. Clin. N. Am. Food Anim. Pract..

[3] Foley J.A., Otterby D.E. (1978). Availability, Storage, Treatment, Composition, and Feeding Value of Surplus Colostrum: A Review. J. Dairy Sci..

[4] DeNise S.K., Robison J.D., Stott G.H., Armstrong D.V. (1989). Effects of Passive Immunity on Subsequent Production in Dairy Heifers. J. Dairy Sci..

[5] Godden S.M., Lombard J.E., Woolums A.R. (2019). Colostrum Management for Dairy Calves. Vet. Clin. N. Am. Food Anim. Pract..

[6] Lora I., Barberio A., Contiero B., Paparella P., Bonfanti L., Brscic M., Stefani A.L., Gottardo F. (2018). Factors Associated with Passive Immunity Transfer in Dairy Calves: Combined Effect of Delivery Time, Amount and Quality of the First Colostrum Meal. Animal.

[7] Sutter F., Venjakob P.L., Heuwieser W., Borchardt S. (2023). Association between Transfer of Passive Immunity, Health, and Performance of Female Dairy Calves from Birth to Weaning. J. Dairy Sci..

[8] Dewell R.D., Hungerford L.L., Keen J.E., Laegreid W.W., Dee Griffin D., Rupp G.P., Grotelueschen D.M. (2006). Association of Neonatal Serum Immunoglobulin G1 with Health and performance in Beef Calves. Sci. Rep. Orig. Study JAVMA.

[9] Faber S.N., Faber N.E., Mccauley T.C., Ax R.L. (2005). Case Study: Effects of Colostrum Ingestion on Lactational Performance 1. Prof. Anim. Sci..

[10] Crouch C.F., Oliver S., Hearle D.C., Buckley A., Chapman A.J., Francis M.J. (2001). Lactogenic Immunity Following Vaccination of Cattle with Bovine Coronavirus. Vaccine.

[11] Franklin S.T., Newman M.C., Newman K.E., Meek K.I. (2005). Immune Parameters of Dry Cows Fed Mannan Oligosaccharide and Subsequent Transfer of Immunity to Calves. J. Dairy Sci..

[12] Civra A., Altomare A., Francese R., Donalisio M., Aldini G., Lembo D. (2019). Colostrum from Cows Immunized with a Veterinary Vaccine against Bovine Rotavirus Displays Enhanced In Vitro Anti-Human Rotavirus Activity. J. Dairy Sci..

[13] Liebler-Tenorio E., Riedel-Caspari G., Pohlenz J. (2002). Uptake of Colostral Leukocytes in the Intestinal Tract of Newborn Calves. Vet. Immunol. Immunopathol..

[14] Reber A.J., Hippen A.R., Hurley D.J. (2005). Effects of the Ingestion of Whole Colostrum or Cell-Free Colostrum on the Capacity of Leukocytes in Newborn Calves to Stimulate or Respond in One-Way Mixed Leukocyte Cultures. Am. J. Vet. Res..

[15] Riedel-Caspari G. (1993). The Influence of Colostral Leukocytes on the Course of an Experimental *Escherichia coli* Infection and Serum Antibodies in Neonatal Calves. Vet. Immunol. Immunopathol..

[16] Donovan D.D., Reber A.J., Gabbard J.D., Aceves-Avila M., Galland K.L., Holbert K.A., Ely L.O., Hurley D.J. (2007). Effect of Maternal Cells Transferred with Colostrum on Cellular Responses to Pathogen Antigens in Neonatal Calves. Am. J. Vet. Res..

[17] Robbers L., van de Mheen R., Benedictus L., Jorritsma R., Nielen M., Bijkerk H.J.C., van der Grein S.G., Ravesloot L., Koets A.P. (2022). Evidence for Transfer of Maternal Antigen Specific Cellular Immunity against *Mycobacterium avium* subsp. *paratuberculosis* via Colostrum in a Goat Twin Model. Vet. Immunol. Immunopathol..

[18] Tello M., Ocejo M., Oporto B., Lavín J.L., Hurtado A. (2022). Within-Farm Dynamics of ESBL-Producing Escherichia Coli in Dairy Cattle: Resistance Profiles and Molecular Characterization by Long-Read Whole-Genome Sequencing. Front. Microbiol..

[19] Real Decreto 53/2013 (2013). Por el que se Establecen las Normas Básicas Aplicables para la Protección de los Animales Utilizados en Experimentación y Otros Fines Científicos, Incluyendo la Docencia.

[20] McGuirk S.M., Collins M. (2004). Managing the Production, Storage, and Delivery of Colostrum. Vet. Clin. N. Am. Food Anim. Pract..

[21] Lombard J., Urie N., Garry F., Godden S., Quigley J., Earleywine T., McGuirk S., Moore D., Branan M., Chamorro M. (2020). Consensus Recommendations on Calf- and Herd-Level Passive Immunity in Dairy Calves in the United States. J. Dairy Sci..

[22] Deelen S.M., Ollivett T.L., Haines D.M., Leslie K.E. (2014). Evaluation of a Brix Refractometer to Estimate Serum Immunoglobulin G Concentration in Neonatal Dairy Calves. J. Dairy Sci..

[23] Bielmann V., Gillan J., Perkins N.R., Skidmore A.L., Godden S., Leslie K.E. (2010). An Evaluation of Brix Refractometry Instruments for Measurement of Colostrum Quality in Dairy Cattle. J. Dairy Sci..

[24] Quigley J.D., Lago A., Chapman C., Erickson P., Polo J. (2013). Evaluation of the Brix Refractometer to Estimate Immunoglobulin G Concentration in Bovine Colostrum. J. Dairy Sci..

[25] Elsohaby I., McClure J.T., Waite L.A., Cameron M., Heider L.C., Keefe G.P. (2019). Using Serum and Plasma Samples to Assess Failure of Transfer of Passive Immunity in Dairy Calves. J. Dairy Sci..

[26] Morrill K.M., Conrad E., Lago A., Campbell J., Quigley J., Tyler H. (2012). Nationwide Evaluation of Quality and Composition of Colostrum on Dairy Farms in the United States. J. Dairy Sci..

[27] Godden S. (2008). Colostrum Management for Dairy Calves. Vet. Clin. N. Am. Food Anim. Pract..

[28] Moore M., Tyler J.W., Chigerwe M., Dawes M.E., Middleton J.R. (2005). Effect of Delayed Colostrum Collection on Colostral IgG Concentration in Dairy Cows. J. Am. Vet. Med. Assoc..

[29] Claudia Casagrande A., Carolina Machado G.P., Lucas Rebelatto Brunetto A., Vedovatto M., Miotto Galli G., Schafer Da Silva A. (2022). Cow Milk or Milk Replacer in the Diet of Holstein Calves: Effects on Complete Blood Count, Biochemistry Variables, and Performance. Animal Nutrition.

[30] Wellert S., Hartschuh J. Feeding Milk Replacer Versus Whole Milk. https://dairy.osu.edu/sites/dairy/files/imce/DIBS/DIBS%2039%20Feeding%20Milk%20Replacer%20Versus%20Whole%20Milk%2039-20.pdf.

[31] Juste R.A., Geijo M.V., Elguezabal N., Sevilla I.A., Alonso-Hearn M., Garrido J.M. (2021). Paratuberculosis Vaccination Specific and Non-Specific Effects on Cattle Lifespan. Vaccine.

[32] Yan J., Nielsen T.B., Lu P., Talyansky Y., Slarve M., Reza H., Novakovic B., Netea M.G., Keller A.E., Warren T. (2023). A Protein-Free Vaccine Stimulates Innate Immunity and Protects against Nosocomial Pathogens. Sci. Transl. Med..

[33] Coffman R.L., Sher A., Seder R.A. (2010). Vaccine Adjuvants: Putting Innate Immunity to Work. Immunity.

[34] Brandon M.R., Watson D.L., Lascelles A.K. (1971). The Mechanism of Transfer of Immunoglobulin into Mammary Secretion of Cows. Aust. J. Exp. Biol. Med. Sci..

[35] Dunn A., Ashfield A., Earley B., Welsh M., Gordon A., Morrison S.J. (2017). Evaluation of Factors Associated with Immunoglobulin G, Fat, Protein, and Lactose Concentrations in Bovine Colostrum and Colostrum Management Practices in Grassland-Based Dairy Systems in Northern Ireland. J. Dairy Sci..

[36] Hagiwara K., Domi M., Ando J. (2008). Bovine Colostral CD8-Positive Cells Are Potent IFN-γ-Producing Cells. Vet. Immunol. Immunopathol..

[37] Myles I.A., Datta S.K. (2021). Frontline Science: Breast Milk Confers Passive Cellular Immunity via CD8-Dependent Mechanisms. J. Leukoc. Biol..

[38] Chien Y.-H., Jores R., Crowley M.P. (1996). Recognition by g/δ T Cells. Annu. Rev. Immunol..

[39] Chase C.C.L., Hurley D.J., Reber A.J. (2008). Neonatal Immune Development in the Calf and Its Impact on Vaccine Response. Vet. Clin. N. Am. Food Anim. Pract..

[40] Krueger L.A., Reinhardt T.A., Beitz D.C., Stuart R.L., Stabel J.R. (2016). Effects of Fractionated Colostrum Replacer and Vitamins A, D, and E on Haptoglobin and Clinical Health in Neonatal Holstein Calves Challenged with *Mycobacterium avium* subsp. *paratuberculosis*. J. Dairy Sci..

[41] Ocejo M., Oporto B., Lavín J.L., Hurtado A. (2023). Monitoring Within-Farm Transmission Dynamics of Antimicrobial-Resistant *Campylobacter* in Dairy Cattle Using Broth Microdilution and Long-Read Whole Genome Sequencing. Sci. Rep..

[42] Ocejo M., Mugica M., Oporto B., Lavín J.L., Hurtado A. (2024). Whole-Genome Long-Read Sequencing to Unveil *Enterococcus* Antimicrobial Resistance in Dairy Cattle Farms Exposed a Widespread Occurrence of *Enterococcus lactis*. Microbiol. Spectr..

