# Supplementary material for: A Pilot Study on Management Practices in Dairy Farms in the Basque Country: Focus on Colostrum Feeding and Vaccination
Source: Animals (Basel). 2025 May 6;15(9):1336. doi: 10.3390/ani15091336 (PMC12071018; doi:10.3390/ani15091336)
Supplement: Supplementary file 1 [file animals-15-01336-s001.zip › animals-3594427-supplementary.pdf]

Supplementary Materials

Information S1. Colostrum Management Guidelines

Farmers were asked to adhere to the following colostrum management recommendations:

1.1 Wash all the equipment and recipients used for colostrum with hot water and soap to guarantee the complete elimination of fat that can contribute to bacterial contamination with a high probability.

1.2 Colostrum milking should be done as soon as possible, and near the feeding time to avoid the loss of immunoglobulins and prevent contamination.

1.3 If possible, the colostrum should not be pooled to avoid diluting the IgG concentration.

1.4 Estimate the IgG concentration using Brix digital refractometer before colostrum administration.

1.5 Adapt the volume of colostrum administered depending on the obtained Brix% results following the table provided below.

1.6 Administer colostrum with values at least over 17% of Brix value.

1.7 Feed with fresh colostrum by bottle nipple or oesophageal tube.

1.8 The first feeding must take place in the first 6 hours after birth and the rest of the volume must be given in 3 feedings, and when possible, twice a day for 2-3 days more.

Table S1. Colostrum total volume feeding adaptation in the first 12 hours and the successive feedings depending on the values obtained by Brix refractometry.

Brix Refractometer Value (%)	Total Volume (Liters in the First 12 h; First Two Feedings)	Total Volume (Liters During 12-24 h; Successive Feedings)
>21	4	2
20-19	4,5	2,5
18-17	5	3

Table S2. Colostrum, feeding, and dry period management in farms.

Farm	Total Number of Heads	Number of Milked Heads	Colostrum Extraction Time	Colostrum Feeding Days; Liters; Feedings/Day	Colostrum Origin	Feeding Until Weaning	Weaning Age (Days)	Dry Period (Days)/ Treatment
A	310	200	ordinary milking time	3; 2.5; 2	Mixture from recently calfed dams	Milk replacer	60	45-60/ blanket
B	140	75	shortly after parturition	1.5; 2; 2	From a multiparous dam own or not	Farm milk	70	45/ selective
C	240	100	ordinary milking time	2; 2 2	From own dam	Milk replacer	60	45/ blanket
D	250	120	shortly after parturition	1.5; 2; 2	From a multiparous dam own or not	Milk replacer	70	60/ blanket

Table S3. Vaccination programs run on farms.

Farm	Diseases	Vaccination Scheme
A	Infectious bovine rhinotracheitis	Primary vaccination at 6 months of age, and revaccination one month after and yearly during the dry period.
	Parainfluenza, Bovine respiratory syndrome, respiratory disease y *Mannheimia haemolytica*	Yearly during the dry period.
	Clostridiosis	Yearly during the dry period.
	Mastitis by *Streptococcus* spp, *Staphylococcus aureus* and *Escherichia coli* strains	Heifer: Primary vaccination at 18 months of age and revaccination at 24 months of age.<br>Lactating cows: revaccination yearly during the dry period.
B	Parainfluenza, Bovine respiratory syndrome, respiratory disease y *Mannheimia haemolytica*	Yearly during the dry period.
	Neonatal diarrhea	Yearly during the dry period.
	Clostridiosis	Blanket vaccination in March in animals over 3 months of age.
	Bluetongue virus	Yearly during the dry period.
C	None	No vaccination is performed.
D	Infectious bovine rhinotracheitis	Blanket vaccination every 6 months.
	Bovine viral diarrhea	Yearly blanket vaccination.
	Clostridiosis	Blanket vaccination every 6 months.

Table S4. IgG estimation in serum.

Farm	Calf	Serum Brix% Values	Serum IgG (mg/mL)
A	1	7	3.33
A	2	9.3	3.33
A	3	7.4	10.42
A	4	8	3.86
A	5	7.4	6.02
A	6	9.2	7.62
A	7	8.1	5.90
B	1	8	22.50
B	2	11.3	27.33
B	3	10.6	49.82
B	4	9.5	30.31
B	5	10.01	37.60
B	6	10	17.40
B	7	8.7	17.40
B	8	8.3	10.17
C	1	10.6	24.08
C	2	9.1	8.67
C	3	8.7	7.28
C	4	10.4	35.75
C	5	9.5	9.98
C	6	8.9	13.45
C	7	10.8	38.54
C	8	8.6	21.94
C	9	6.6	2.67
C	10	10.4	19.62
D	1	10.4	19.36
D	2	7.9	6.02
D	3	8.2	7.45
D	4	8.3	14.75
D	5	8.7	37.60
D	6	7.1	2.35
D	7	9.4	13.24
